# Supplementary material for: Association between First-Generation Antihistamine Use in Children and Cardiac Arrhythmia and Ischemic Heart Disease: A Case-Crossover Study
Source: Pharmaceuticals (Basel). 2023 Jul 28;16(8):1073. doi: 10.3390/ph16081073 (PMC10457948; doi:10.3390/ph16081073)
Supplement: Supplementary file 1 [file pharmaceuticals-16-01073-s001.zip › pharmaceuticals-2477723-supplementary.pdf]

Supplementary Table S1. Risk of Cardiovascular Events Associated with the Use of First-Generation Antihistamine in Children with Respiratory Infection[a].

Time window before the cardiovascular events	Total N	N (%)			Crude OR (95% CI)	Adjusted OR (95% CI) [b]
		Exposed during the hazard period	Exposed during the control period 1	Exposed during the control period 2		
0–15 days	1,860	945 (50.8)	756 (40.6)	728 (39.1)	**1.192 (1.127-1.261)**	**1.197 (1.126-1.272)**
0–10 days	1,622	814 (50.2)	616 (38.0)	593 (36.6)	**1.229 (1.157-1.305)**	**1.250 (1.169-1.337)**
0–5 days	1,217	592 (48.6)	432 (35.5)	426 (35.0)	**1.229 (1.146-1.317)**	**1.236 (1.141-1.340)**

Abbreviations, N, number; OR, odd ratio; CI confidence interval;

[a] Respiratory infection was defined as acute upper respiratory infection, influenza, pneumonia, or acute lower respiratory infection, and participants were limited to those diagnosed with respiratory infection in the hazard period or control periods.

[b] Adjusted for sex, age, residence area, economic status, season, comorbidities, concomitant disease, and concomitant medication use.

Supplementary Table S2. Checklist of Recommendations for Reporting of Observational Studies Using the Reporting of Studies Conducted Using Observational Routinely Collected Health Data (RECORD) Guidelines

	Item No	Recommendation	Reported
Title and abstract	1	(a) Indicate the study's design with a commonly used term in the title or the abstract	Abstract
		(b) Provide in the abstract an informative and balanced summary of what was done and what was found	Abstract
**Introduction**			
Background /rationale	2	Explain the scientific background and rationale for the investigation being reported	Introduction
Objectives	3	State specific objectives, including any prespecified hypotheses	Introduction
**Methods**			
Study design	4	Present key elements of study design early in the paper	Methods – case cross-over study design and Figure 2
Setting	5	Describe the setting, locations, and relevant dates, including periods of recruitment, exposure, follow-up, and data collection	Methods – data source and participants
Participants	6	(a) Give the eligibility criteria, and the sources and methods of selection of participants. Describe methods of follow-up	Methods – data source, participants and Figure 1
		(b) For matched studies, give matching criteria and number of exposed and unexposed	NA
Variables	7	Clearly define all outcomes, exposures, predictors, potential confounders, and effect modifiers. Give diagnostic criteria, if applicable	Methods – cardiovascular events and 1st generation H1 antihistamine use
Data sources /measurement	8	For each variable of interest, give sources of data and details of methods of assessment (measurement). Describe comparability of assessment methods if there is more than one group	Methods – data source and covariates
Bias	9	Describe any efforts to address potential sources of bias	Methods – Covariates; Statistical analysis;
Study size	10	Explain how the study size was arrived at	Methods – participants and Figure 1
Quantitative variables	11	Explain how quantitative variables were handled in the analyses. If applicable, describe which groupings were chosen and why	Methods – statistical analysis
Statistical methods	12	(a) Describe all statistical methods, including those used to control for confounding	Methods – Covariates; statistical analysis
		(b) Describe any methods used to examine subgroups and interactions	Methods – statistical analysis
		(c) Explain how missing data were addressed	Statistical analysis and Table 1

		(d) If applicable, explain how loss to follow-up was addressed	NA
		(e) Describe any sensitivity analyses	Methods – Study population; Statistical analysis
**Results**			
Participants	13	(a) Report numbers of individuals at each stage of study, e.g., numbers potentially eligible, examined for eligibility, confirmed eligible, included in the study, completing follow-up, and analyzed	Results – Participants and Figure 1
		(b) Give reasons for non-participation at each stage	Figure 1
		(c) Consider use of a flow diagram	Figure 1
Descriptive data	14	(a) Give characteristics of study participants (e.g. demographic, clinical, social) and information on exposures and potential confounders	Results – Participants and covariates, Table 1
		(b) Indicate number of participants with missing data for each variable of interest	Methods – Table 1
		(c) Summarize follow-up time (e.g. average and total amount)	Methods – Data sources
Outcome data	15	Report numbers of outcome events or summary measures over time	Results – Table 2, and eTable 5
Main results	16	(a) Give unadjusted estimates and, if applicable, confounder-adjusted estimates and their precision (e.g., 95% confidence interval). Make clear which confounders were adjusted for and why they were included	Results – Table 2 and eTable 5
		(b) Report category boundaries when continuous variables were categorized	Not applicable
		(c) If relevant, consider translating estimates of relative risk into absolute risk for a meaningful time period	Not applicable
Other analyses	17	Report other analyses done, e.g., analyses of subgroups and interactions, and sensitivity analyses	Results – Table 2 and eTable 5
**Discussion**			
Key result	18	Summarize key results with reference to study objectives	Discussion
Limitation	19	Discuss limitations of the study, taking into account sources of potential bias or imprecision. Discuss both direction and magnitude of any potential bias	Discussion
Interpretation	20	Give a cautious overall interpretation of results considering objectives, limitations, multiplicity of analyses, results from similar studies, and other relevant evidence	Discussion
Generalizability	21	Discuss the generalizability (external validity) of the study results	Discussion
Other information			
Funding	22	Give the source of funding and the role of the funders for the present study and, if applicable, for the original study on which the present article is based	Article Information

Supplementary Table S3. Literature Search

1	"histamine antagonists/adverse effects"[MeSH Terms]	3,283
2	"arrhythmias, cardiac"[MeSH Terms] OR ("arrhythmias"[All Fields] AND "cardiac"[All Fields]) OR "cardiac arrhythmias"[All Fields] OR "arrythmia"[All Fields] OR "arrythmias"[All Fields]	243,135
3	1 AND 2	235
4	"arrhythmias, cardiac"[MeSH Terms]	226,326
5	1 AND 4	223
6	[1 AND 2] OR [1 AND 4] AND (humans[Filter])	216
7	6 AND (allchild[Filter])	30

Supplementary Table S4. Summary of Previous Studies on H1-antihistamine-Induced Cardiovascular Toxicity in Children

Author/year of publication	Type of study	Age/sex	Drug	Route	Cardiovascular events
Tenley and Friedman[1] 1966	Case-report	2 months/male	Methapyrilene hydrochloride	Percutaneous	Complete heart block with complete atrioventricular dissociation
Magera et al[2] 1981	Case-report	13 months/female	Hydroxyzine overdose	Per oral	Sinus tachycardia with generalized seizure
Craft[3] 1986	Case-report	16 years/female	Astemizole overdose	Per oral	Torsade de pointes
Snook et al [4] 1988	Case-report	17 months/male	Astemizole overdose		Multiform PVCs, brief salvoes of VT, torsades de pointes
Simons et al[5] 1988	Case-report	15 years/female	Astemizole	Per oral	Torsade de pointes
Bishop and Gaudry[6] 1989	Case-report	16 years/female	Astemizole overdose	Per oral	Sinus tachycardia with a prolongation of the QT interval
Hoppu et at[7] 1991	Short communication	1 year 7 months/male<br>2 years 6 months/male<br>2 years 4 months/female<br>2 years/male<br>3 years 4 years/male<br>2 years/male	Astemizole overdose		Prolongation of the QT interval
Heidemann and Sarnaik[8] 1996	Case-report	15 years/female<br>11 months/female	Astemizole overdose	Per oral	Asystole, ventricular fibrillation, QT prolongation
Wong and Rasool[9] 2004	Case-report	9 year/female	Hydroxyzine	Per oral	Supraventricular tachycardia
Jeffrey et al[10] 2010	Review	1. 34 months/female<br>2. 15 months/ male<br>3. 14 years/female	Diphenhydramine		1. Monophasic wide complex tachycardia<br>2. Tachycardia<br>3. Wide complex idioventricular bradycardia

		4. 9 weeks/4.5 kg<br>5. 17 years/male<br>6. 18 years/female			4. Asystole<br>5. Death<br>6. Death
Paudel et al[11] 2011	Case-report	15 years/female	Pyrilamine overdose	Per oral	Prolongation of QT interval
Alice et al[12] 2017	Case-report	Fetus, gestational age 41 weeks	Diphenhydramine	Intravenous to mother	Tachycardia
**Author/year of publication**	**Type of study**	**Design**		**Outcome**	**Results**
Hanrahan et al[13] 1995	Observational study	Terfenadine (9,008) vs other H1-antihistamine (13,344)		Clinical/arrhythmia event<br>QT prolongation	OR (95% CI)<br>0.86 (0.52-1.44)<br>1.00 (0.64-1.57)
Staffa et al[14] 1995	Observational study	Astemizole (15,585) vs sedating H1-antihistamine (30,105)		Ventricular arrhythmia and sudden death	RR (95% CI)<br>0.25 (0.11-0.58)
Pesco-Koplowitz [15] et al 1999	Two randomized, open-label, placebo-controlled, two-way crossover studies	Levocabastine (intranasal spray) plus erythromycin or ketoconazole vs levocabastine plus placebo		QT interval<br>EKG	No change in QT interval
Abajo et al[16] 2001	a cohort study with a nested case-control analysis	Nonsedating H1 antihistamine use vs non use		Idiopathic ventricular arrhythmias	RR (95% CI)<br>4.2 (1.5-11.8)
Bruin et al[17] 2002	Observational study	Nonsedating H1 antihistamine use vs other drugs		Heart rate and rhythm disorders	OR (95% CI)<br>2.05 (1.45-2.89)
Laforest et al[18] 2007	Cross-sectional study	Questionnaire of asthma patients with H1 antihistamine		Adverse events	Did not affect reporting adverse events suggesting that adverse events were mainly due to asthma therapy or other causes.
Hulhoven et al[19] 2007	placebo and positive controlled, four-way crossover randomized trial	Levocetirizine vs placebo vs moxifloxacin		ΔQTcSS	Levocetirizine 5 mg<br>= 2.86 (90% CI, 0.02-5.7)

				Levocetirizine 30 mg<br><br>= 1.06 (90% CI, -1.78-3.9)<br><br>Moxifloxacin 400 mg<br><br>= 13.37 (90% CI, 10.53-16.21)<br><br>Levocetirizine does not prolong the QT/QTc interval in healthy participants.
Leonid et al[20] 2010	Randomized controlled pilot trial	Quifenadine vs amiodarone in children with frequent premature beats	Full antiarrhythmic efficacy (PB <75% from baseline)	43% vs 74%, *P* = 0.02<br><br>Side effect = 2% vs 40%
Cantrell et al[21] 2015	Retrospective observational study	140 cases of doxylamine overdose in a poison system database	Symptoms	Tachycardia, 3 cases (over 6.2 mg/kg doxylamine)
Vries and Hunsel[22] 2016	Retrospective observational study	Adverse drug reactions on systemic antihistamines reported to the Netherlands Pharmacovigilance Centre Lareb in 1991–2014	Adverse drug reactions	One death (malignant neuroleptic syndrome), cardiac arrhythmia (one case) and convulsions (three cases).
Ali et al[23] 2021	Retrospective observational study	Adverse events reported to the FARES database	Torsade de Pointes	Reporting odds ratio (95% CI)<br><br>Cetirizine = 3.2 (2.2–4.6)<br><br>Chlorpheniramine = 12.6 (98.1-19.6)<br><br>Clemastine = 61.4 (42.5–88.8)<br><br>Desloratadine = 4.3 (1.8–10.3)<br><br>Loratadine = 7.1 (5.6–8.9)<br><br>Meclizine = 7.4 (2.8–19.7)<br><br>Astemizole = 31.4 (20.4–48.3)<br><br>Diphenhydramine = 8.4 (6.5–10.8)<br><br>Hydroxyzine = 16.7 (13.2–21.1)<br><br>Terfenadine = 25.5 (19.7–33.0)<br><br>Trimeprazine = 12.9 (6.4–25.8)

Abbreviations, CI, confidence interval; RR, risk ratio; EKG, electrocardiogram; PVCs, premature ventricular contractions; VT, ventricular tachycardia; OR, odd ratio; PB premature beat.

Supplementary Table S5. Coding Definitions for Demographic and Comorbid Conditions

Characteristic	Database	Codes or definition
**Demographics**		
Age	NHIS	
Gender	NHIS	
Residence area	NHIS	
Economic status	NHIS	
Birth weight	NHSPIC	
**Clinical**		
Season at index date	NHIS	
Comorbidities		
Kawasaki disease	NHIS, ICD-10 code	M 30.3
Obesity	NHSPIC	BMI z score >1.645
Sleep apnea	NHIS, ICD-10 code	G47.3
Hyperthyroidism	NHIS, ICD-10 code	E05.X
Diabetes mellitus	NHIS, ICD-10 code	E10.X
Concomitant disease		
Acute nasopharyngitis	NHIS, ICD-10 code	J00.0
Acute tonsillitis	NHIS, ICD-10 code	J03.9
Acute pharyngitis	NHIS, ICD-10 code	J02.9
Acute upper respiratory infection	NHIS, ICD-10 code	J06.9
Acute sinusitis	NHIS, ICD-10 code	J01.9
Acute suppurative otitis media	NHIS, ICD-10 code	H66.0
Acute bronchitis	NHIS, ICD-10 code	J20.9
Acute bronchiolitis	NHIS, ICD-10 code	J21.9
Gastroenteritis and colitis	NHIS, ICD-10 code	A09.0
Allergic rhinitis	NHIS, ICD-10 code	J30.4
Concomitant medication		
Second-generation H1-antihistamine	NHIS, drug classification code	141, 149, 222
Nasal decongestant	NHIS, drug classification code	132
Systemic steroid	NHIS, drug classification code	245

Abbreviations: NHIS, National Health Insurance System; NHSPIC, National Health Screening Program for Infants and Children; ICD, International Classification of Diseases; BMI, body mass index.

REFERENCES

1. Tenley JA, Friedman S. A toxic cardiac reaction to drugs absorbed percutaneously. *J Pediatr*. 1966;69(2):299-301.
2. Magera BE, Betlach CJ, Sweatt AP, Derrick Jr CW. Hydroxyzine intoxication in a 13-month-old child. *Pediatrics*. 1981;67(2):280-283.
3. Craft TM. Torsade de pointes after astemizole overdose. *Br Med J (Clin Res Ed)*. 1986;292(6521):660.
4. Snook J. Torsade de pointes ventricular tachycardia associated with astemizole overdose. *Br J Clin Pract*. 1988;42:257-259.
5. Simons F, Kesselman M, Giddins N, Pelech AN, Simons K. Astemizole-induced torsade de pointes. *Lancet*. 1988;332(8611):624.
6. Bishop R, Gaudry P. Prolonged QT interval following astemizole overdose. *Emerg Med J*. 1989;6(1):63-65.
7. Hoppu K, Tikanoja T, Tapanainen P, Remes M, Kouvalainen K, Saarenpää O. Accidental astemizole overdose in young children. *Lancet*. 1991;338(8766):538-540.
8. Heidemann SM, Sarnaik AP. Arrhythmias after astemizole overdose. *Pediatr Emerg Care*. 1996;12(2):102-104.
9. Wong A, Rasool A. Hydroxyzine-induced supraventricular tachycardia in a nine-year-old child. *Singapore Med J*. 2004;45(2):90-92.
10. Kuffner E, Patel M. Fatality from diphenhydramine monointoxication: a case report and review of the infant, pediatric, and adult literature. *Am J Forensic Med Pathol*. 2010;31(1):106.
11. Paudel G, Syed M, Kalantre S, Sharma J. Pyrilamine-induced prolonged QT interval in adolescent with drug overdose. *Pediatr Emerg Care*. 2011;27(10):945-947.
12. Abernathy A, Alsina L, Greer J, Egerman R. Transient fetal tachycardia after intravenous diphenhydramine administration. *Obstet Gynecol*. 2017;130(2):374-376.
13. Hanrahan JP, Choo PW, Carlson W, Greineder D, Faich GA, Platt R. Terfenadine-associated ventricular arrhythmias and QTc interval prolongation: a retrospective cohort comparison with other antihistamines among members of a health maintenance organization. *Ann Epidemiol*. 1995;5(3):201-209.
14. Staffa JA, Jones JK, Gable CB, Verspeelt JP, Amery WK. Risk of selected serious cardiac events among new users of antihistamines. *Clin Ther*. 1995;17(6):1062-1077.
15. Pesco-Koplowitz L, Hassell A, Lee P, Zhou H, Hall N, Wiesinger B, et al. Lack of Effect of Erythromycin and Ketoconazole on the Pharmacokinetics and Pharmacodynamics of Steady-State Intranasal Levocabastine. *J Clin Pharmacol*. 1999;39(1):76-85.
16. José de Abajo F, Rodríguez LAG. Risk of ventricular arrhythmias associated with nonsedating antihistamine drugs. *British journal of clinical pharmacology*. 1999;47(3):307-313.
17. De Bruin ML, Van Puijenbroek EP, Egberts A, Hoes AW, Leufkens HG. Non-sedating antihistamine drugs and cardiac arrhythmias–biased risk estimates from spontaneous reporting systems? *Br J Clin Pharmacol*. 2002;53(4):370-374.
18. Laforest L, Van Ganse E, Devouassoux G, Osman LM, Bauguil G, Chamba G. Patient-reported Adverse Events Under Asthma Therapy: A Community Pharmacy-based Survey. *Clin Pharmacol Ther*. 2007;82(2):167-172.

19. Hulhoven R, Rosillon D, Letiexhe M, Meeus M-A, Daoust A, Stockis A. Levocetirizine does not prolong the QT/QTc interval in healthy subjects: results from a thorough QT study. *Eur J Clin Pharmacol*. 2007;63(11):1011-1017.

20. Makarov L, Balykova L, Soldatova O, Komolyatova V, Serebruany V. The antiarrhythmic properties of Quifenadine, H1-histamine receptor blocker in children with premature beats: A randomized controlled pilot trial. *Am J Ther*. 2010;17(4):396-401.

21. Cantrell FL, Clark AK, McKinley M, Qozi M. Retrospective review of unintentional pediatric ingestions of doxylamine. *Clin Toxicol*. 2015;53(3):178-180.

22. de Vries TW, van Hunsel F. Adverse drug reactions of systemic antihistamines in children in the Netherlands. *Arch Dis Child*. 2016;101(10):968-970.

23. Ali Z, Ismail M, Khan F, Sajid H. Association of H1-antihistamines with torsade de pointes: a pharmacovigilance study of the food and drug administration adverse event reporting system. *Expert Opin Drug Saf*. 2021;20(1):101-108.
